# I-131 false-positive uptake in a thymic cyst with expression of the sodium-iodide symporter: A case report

**DOI:** 10.1097/MD.0000000000029282

**Published:** 2022-06-30

**Authors:** Erina Yano, Masatoyo Nakajo, Megumi Jinguji, Atsushi Tani, Ikumi Kitazono, Takashi Yoshiura

**Affiliations:** a Department of Radiology, Kagoshima University Graduate School of Medical and Dental Sciences, Kagoshima, Japan; b Department of Pathology, Kagoshima University Graduate School of Medical and Dental Sciences, Kagoshima, Japan.

**Keywords:** I-131 radioiodine therapy, NIS, thymic cyst, thyroid cancer, whole body scintigraphy

## Abstract

I-131 radioiodine false-positive findings in postoperative patients with differentiated thyroid cancer (DTC) should be recognized to avoid unnecessary therapies.

**Patient concerns and diagnoses:**

A 50-year-old man underwent I-131 therapy 3 times, including the initial ablative therapy after total thyroidectomy for papillary thyroid cancer. The initial I-131 posttherapeutic whole-body scintigraphy showed 2 cervical and one superior mediastinal focal I-131 positive uptake lesions. The serum thyroglobulin level was negative every time when the radioiodine therapy was performed. Although the 2 cervical positive uptake lesions disappeared after the second therapy, the superior mediastinal I-131 positive uptake persisted even after the third therapy, and this lesion was suspicion of I-131 therapy-resistant node metastasis.

**Interventions and outcomes:**

The lesion was resected, and the pathological diagnosis with immune-histochemical analysis was a thymic cyst with thymic epithelial cells having a weak expression of the sodium-iodide symporter (NIS).

**Lessons:**

The false-positive result may be attributed to the NIS expression in the thymic cyst epithelial cells. It is necessary to include a thymic cyst in the differential diagnosis, when I-131 uptake is noted in the superior mediastinal region on I-131 posttherapeutic scans of patients with postoperative DTC. Although the I-131 positive uptake in a thymic cyst may be influenced by the I-131 administered dose and scan timing after I-131 administration, the NIS expression may be essential to the false-positive uptake in a thymic cyst.

## 1. Introduction

Differentiated thyroid cancers (DTCs) are usually managed by total or near-total thyroidectomy followed by I-131 ablation of any remnant thyroid tissue.^[1]^ On I-131 whole body scintigraphy (WBS), with the exception of physiological radioiodine uptake in the salivary glands, stomach, and gastrointestinal and urinary tracts, lesions exhibiting radioiodine uptake can be considered to be metastatic in thyroid cancer patients who have previously undergone total thyroidectomy.^[2,3]^ However, there are many causes that may give rise to I-131 false-positive WBS in postoperative patients with DTC^[4]^; therefore, it is imperative to carefully evaluate abnormal scans in order to appropriately manage patients with DTC. The present case describes an I-131 false-positive uptake in a thymic cyst with a weak expression of sodium-iodide symporter (NIS) in thymic epithelial cells. The thymic cyst should be kept in mind as one of the possible false-positive findings on I-131 WBS to avoid unnecessary therapies.

## 2. Case presentation

**Figure 1.**
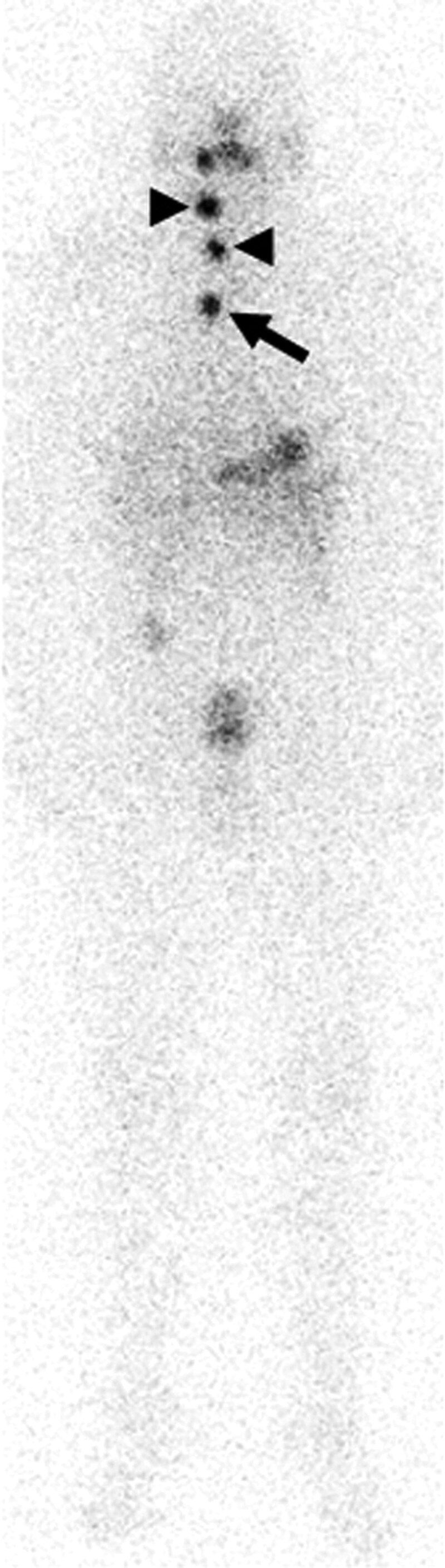
A 50-year-old man received an ablative I-131 dose after total thyroidectomy for papillary thyroid cancer. I-131 whole body scintigraphy (WBS) 3 days after the ablative therapy shows 2 positive cervical nodular lesions (arrowheads) and a focal positive nodular lesion in the superior mediastinum (arrow).

A 50-year-old man presented to the private hospital with an incidentally detected nodule in the right thyroid lobe. A fine needle aspiration revealed papillary thyroid cancer. Subsequently, he was referred to our hospital and underwent total thyroidectomy with neck dissection. Gross and microscopic examinations disclosed a 26 × 22 × 18 mm papillary carcinoma in the right thyroid lobe with metastatic lymph nodes. There was no extrathyroidal extension (pT2N1bM0, stage II). Thereafter, he was referred to our department for remnant ablation. Three months after surgery, he received an ablative I-131 dose of 30 mCi (1.1 GBq) with recombinant thyroid stimulating hormone (TSH). The serum TSH (s-TSH) level was 133.6 ulU/ml (normal range, 0.3–5.0 ulU/ml), and the serum thyroglobulin (s-thyroglobulin) level was 0.2 ng/ml (normal range, <33.7 ng/ml) with negative s-thyroglobulin antibody on the day of I-131 administration. I-131 WBS (Prism 2000; Picker, Shimazu, Japan) 3 days after the administration of I-131 showed 2 positive cervical nodular lesions (Fig. 1, arrowheads) and a positive nodular lesion in the superior mediastinum (Fig. 1, arrow), leading to suspicion of lymph node metastases of thyroid cancer. Based on these I-131 WBS findings, it was decided to continue with I-131 therapy.

**Figure 2.**
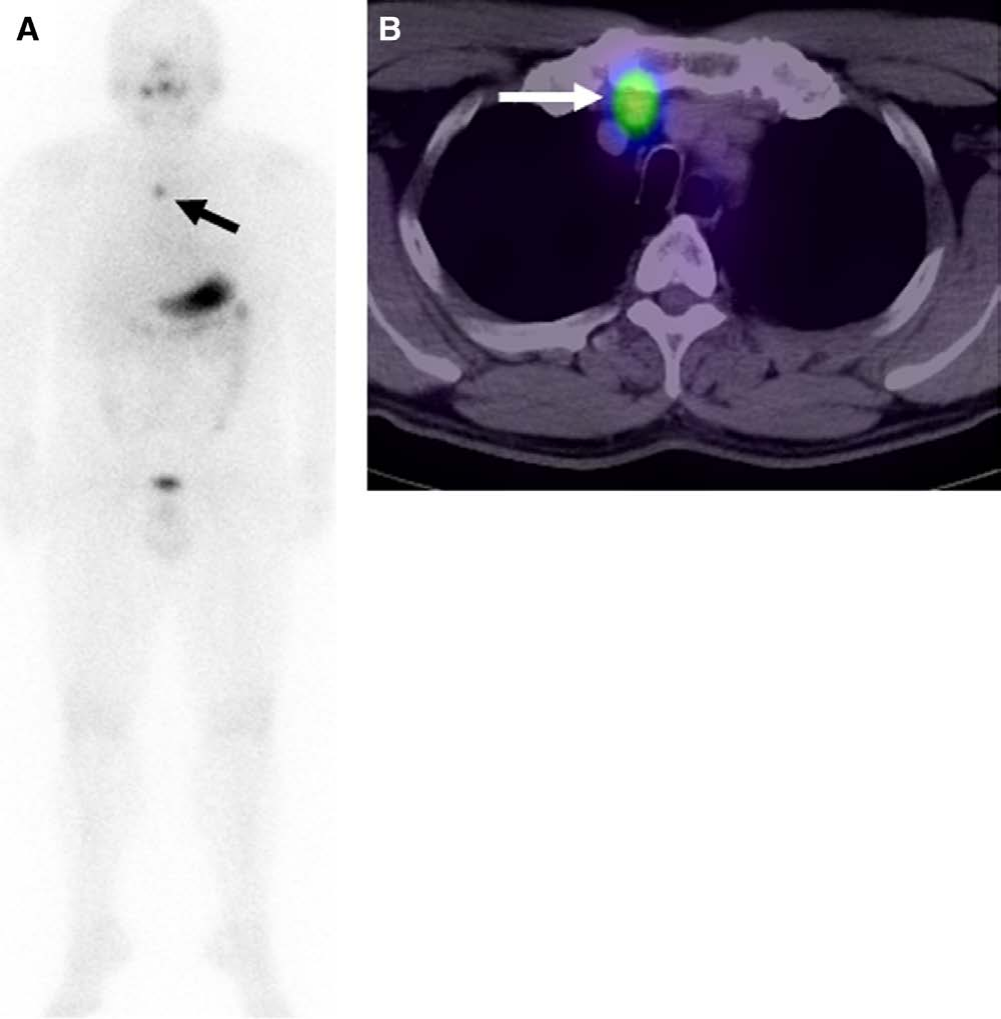
I-131 WBS 3 days after the second therapeutic dose (A) shows the positive uptake disappeared in cervical nodular lesions, but persisted in the superior mediastinum (arrow). I-131 SPECT/CT (B) revealed a hot nodule in the superior mediastinum (arrow).

The second therapy was performed 9 months after the ablative therapy under withdrawal of throxine replacement. The s-TSH level was 209.5 uIU/ml, and the s-thyroglobulin level was 0.1 ng/ml with negative s-thyroglobulin antibody on the day of I-131 administration. On the second I-131 WBS (Siemens Intevo SPECT/CT system; Siemens Medical Solutions USA, Inc., Hoffmann Estates, IL) obtained 3 days after the administration of I-131 of 100 mCi (3.7GBq), the positive uptake disappeared in cervical nodular lesions, but persisted in the superior mediastinum (Fig. 2A, arrow). Single photon emission computed tomography/computed tomography (SPECT/CT) (Siemens Intevo SPECT/CT) images revealed a nodule (Fig. 2B, arrow) with radioiodine uptake in the superior mediastinum. Although s-thyroglobulin was negative, the most suspected diagnosis of this mediastinal nodule was lymph node metastasis. Thus, the third therapy was scheduled 6 months after the second therapy.

**Figure 3.**
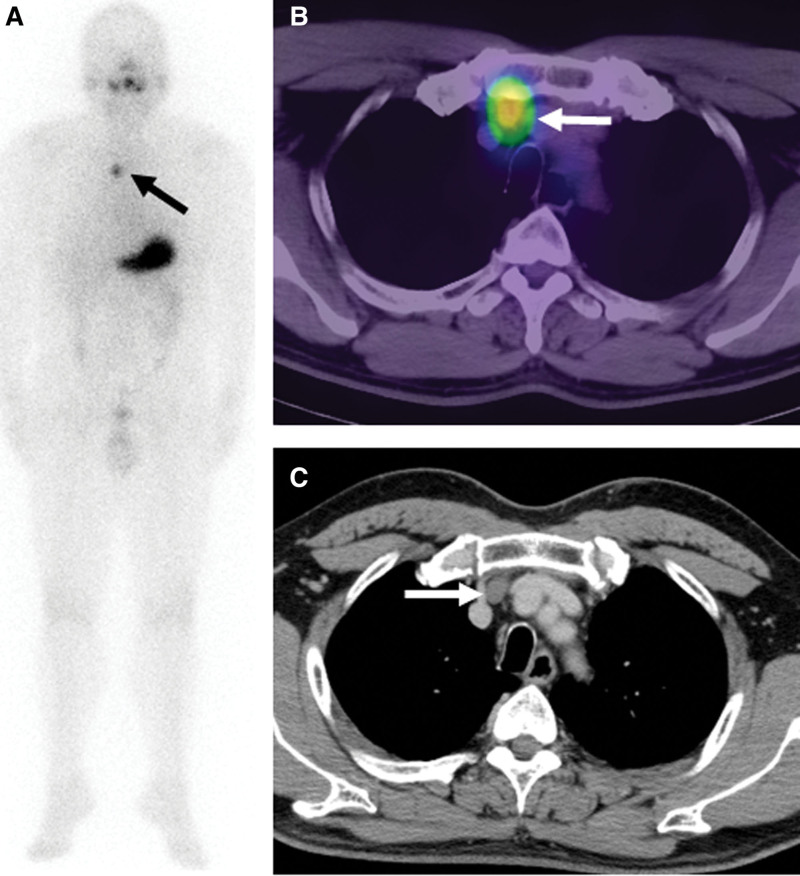
The positive uptake in the superior mediastinal nodule persisted on 131 WBS (A) and I-131 SPECT/CT (B) 3 days after the third therapeutic dose (arrows). Contrast-enhanced CT image (C) shows a nonclear contrast enhanced solid nodule (arrow).

At the third therapy, he received an I-131 dose of 100 mCi (3.7GBq) under withdrawal of throxine replacement. S-thyroglobulin was negative (0.04 ng/ml) with negative s-thyroglobulin antibody, and the TSH level was 250.4 ulU/ml on the day of I-131 administration. The third I-131 WBS 3 days after the administration of I-131 showed the persistent radioiodine uptake in the superior mediastinum (Fig. 3A, arrow). SPECT/CT (Siemens Intevo SPECT/CT system) confirmed the radioiodine uptake in the same mediastinal nodule (Fig. 3B, arrow). On the contrast-enhanced CT image, this appeared as a nonclear contrast enhanced solid nodule (15 mm in diameter, 50.3 Hounsfield unit in density) (Fig. 3C, arrow). Based on these findings, the mediastinal nodule was suspicion of I-131 therapy-resistant node metastasis. Thus, surgical resection of the nodule was planned.

**Figure 4.**
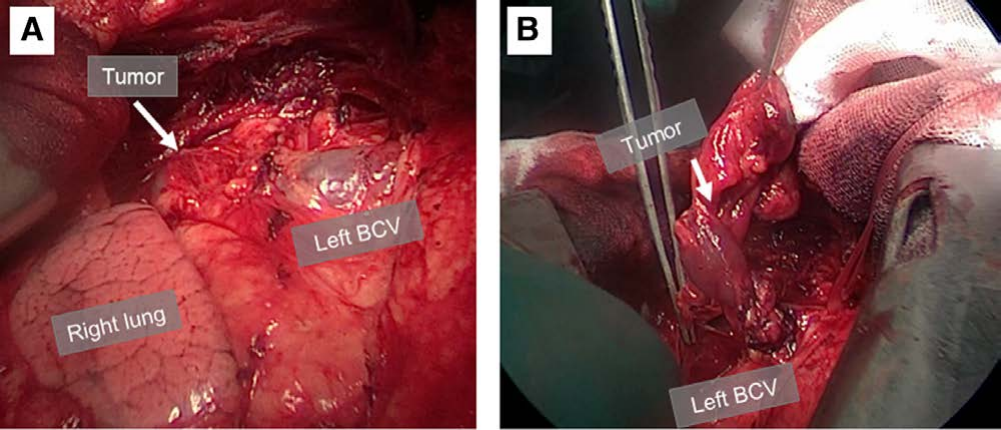
The tumor was resected by median sternotomy. During operation, the tumor (arrows) was adjacent to the left brachiocephalic vein (BCV) (A), and it was easily dissected from the left BCV and removed along with the surrounding adipose tissue (B).

**Figure 5.**
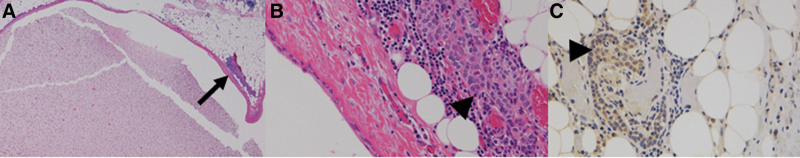
Pathologic examinations of the resected specimen by HE staining (A objective lens x2, B: objective lens x20) and immunohistochemical staining with NIS antibody (C, objective lens x20). Microscopic appearance of the cystic wall (A, arrow) shows thymic epithelial cells (B, arrowhead). Immunohistochemical staining with NIS antibody revealed a weak expression of NIS in thymic epithelial cells (C, arrowhead).

Three months after the third therapy, the nodule was resected by median sternotomy (Fig. 4A, B). Pathologic examination of the resected specimen revealed a tumor with the cystic wall (Fig. 5A, arrow) having thymic epithelial cells (Fig. 5B, arrowhead), resulting in the diagnosis of a thymic cyst. Immunohistochemical staining with NIS antibody revealed a weak expression of NIS in thymic epithelial cells (Fig. 5C, arrowhead).

Three years after total thyroidectomy, the patient is doing well without recurrence.

## 3. Discussion

Thymic cysts are rare mediastinal masses with congenital abnormalities due to persistent and consequential cystic degeneration of the third pharyngeal pouch.^[5]^ To our knowledge, there are 3 case reports of a thymic cyst with I-131 false-positive uptake after I-131 therapy.^[6-8]^ In addition to physiological accumulation in the stomach, salivary gland, and ovary, I-131 false-positive uptake has also been reported in cystic lesions of other organs, such as pleuropericardial cyst. ^[9]^

In our case, the initial I-131 WBS without SPECT/CT images indicated the presence of possible multiple lymph node metastases. The second I-131 WBS with SPECT/CT images showed persistent radioiodine uptake in the superior mediastinal nodule in spite of disappearance of positive uptake in the 2 cervical nodular lesions. Although s-thyroglobulin was negative, the mediastinal lymph node metastasis was suspected at this time. However, the third I-131 WBS with SPECT/CT images also showed the residual radioiodine uptake in the same nodule under negative s-thyroglobulin. Thus, at this time, although I-131 therapy-resistant node metastasis was mostly suspected, some false positive uptake was also suggested. However, we could not include thymic cyst as a differential diagnosis because of rarity of the I-131 false-positive uptake case and atypical CT density (50.3 Hounsfield unit). The mean density of true thymic cysts was 23 Hounsfield unit.^[10]^

Although the NIS expression was observed in human thymic tissues, whose level of NIS mRNA expression is markedly low and 1.5% compared with that in normal thyroid tissues,^[11]^ the exact underlying mechanism of radioiodine positive uptake in thymic cysts is unknown. To our knowledge, there were no reports which investigated the relationship between NIS expression and radioiodine uptake in thymic cyst epithelial cells. In the present case, a weak NIS expression was noted in the thymic cyst epithelial cells, which might have essentially contributed to I-131 positive uptake in the thymic cyst.

One of the 3 reported false-positive thymic cyst cases showed that the thymic cyst was not visualized by the initial diagnostic SPECT/CT 24 hours after I-123 administration of 5.3 mCi (196.1 MBq), while the cyst was visualized on posttreatment SPECT/CT 1 week after I-131 administration of 157 mCi (5.8 GBq).^[8]^ About thymic I-131 false-positive uptake, it was also observed on I-131 posttreatment scans, although a retrospective study revealed a few possible thymic positive uptake cases on diagnostic scans from 66 to 72 hours after an oral I-131dose of 2 mCi (74 MBq).^[12]^ Wilson et al^[13]^ reported a relatively high incidence of thymic I-131 false-positive uptake on scans taken 3 and 7 days posttherapy in 10 of 38 consecutive patients with DTC who each received at least 1 therapy dose of I-131, and thymic I-131 false-positive uptake was observed in 8 of 10 patients only on 7-day scans. Thus, the I-131 posttherapeutic thymic cyst uptake may also be influenced by the I-131 administered dose and the scan timing after I-131 administration, as well as the degree of NIS expression.

## 4. Conclusions

The I-131 positive uptake in a thymic cyst is one of the possible I-131 false-positive findings and it may be partly, but essentially attributed to the NIS expression in the thymic cyst epithelial cells.

### Acknowledgments

The authors would like to thank Dr Masaya Aoki, who belongs to Department of Thoracic Surgery, Kagoshima University, Graduate School of Medical and Dental Sciences, for preparing for the surgical images of thymic cyst.

### Author Contributions

Conceptualization: Erina Yano, Masatoyo Nakajo, Megumi Jinguji.

Data curation: Erina Yano, Masatoyo Nakajo, Megumi Jinguji, Atsushi Tani, Ikumi Kitazono.

Supervision: Takashi Yoshiura.

Writing – original draft: Erina, Yano, Masatoyo Nakajo.

Writing – review and editing: Masatoyo Nakajo, Takashi Yoshiura.
